# Mechanism of Action of Electrospun Chitosan-Based Nanofibers against Meat Spoilage and Pathogenic Bacteria

**DOI:** 10.3390/molecules22040585

**Published:** 2017-04-06

**Authors:** Mounia Arkoun, France Daigle, Marie-Claude Heuzey, Abdellah Ajji

**Affiliations:** 1 CREPEC Department of Chemical Engineering, École Polytechnique de Montréal, P.O. Box 6079, Station Centre-Ville, Montréal, QC H3C 3A7, Canada; mounia.arkoun@polymtl.ca; 2 Department of Microbiology, Infectiology and Immunology, Pavillon Roger-Gaudry, Université de Montréal, C.P. 6128, Centre-ville, Montréal, QC H3C 3J7, Canada; france.daigle@umontreal.ca

**Keywords:** chitosan-based nanofibers, mechanism of action, gram-negative, gram-positive, meat packaging

## Abstract

This study investigates the antibacterial mechanism of action of electrospun chitosan-based nanofibers (CNFs), against *Escherichia coli*, *Salmonella enterica* serovar Typhimurium, *Staphylococcus aureus* and *Listeria innocua*, bacteria frequently involved in food contamination and spoilage. CNFs were prepared by electrospinning of chitosan and poly(ethylene oxide) (PEO) blends. The in vitro antibacterial activity of CNFs was evaluated and the susceptibility/resistance of the selected bacteria toward CNFs was examined. Strain susceptibility was evaluated in terms of bacterial type, cell surface hydrophobicity, and charge density, as well as pathogenicity. The efficiency of CNFs on the preservation and shelf life extension of fresh red meat was also assessed. Our results demonstrate that the antibacterial action of CNFs depends on the protonation of their amino groups, regardless of bacterial type and their mechanism of action was bactericidal rather than bacteriostatic. Results also indicate that bacterial susceptibility was not Gram-dependent but strain-dependent, with non-virulent bacteria showing higher susceptibility at a reduction rate of 99.9%. The susceptibility order was: *E. coli* > *L. innocua* > *S. aureus* > *S.* Typhimurium. Finally, an extension of one week of the shelf life of fresh meat was successfully achieved. These results are promising and of great utility for the potential use of CNFs as bioactive food packaging materials in the food industry, and more specifically in meat quality preservation.

## 1. Introduction

Chitosan, a versatile biopolymer generally of marine origin and obtained through chemical or enzymatic deacetylation of chitin, exhibits powerful antimicrobial potential against a wide range of bacteria, fungi, yeasts, viruses, toxins, and spores [1,2,3,4,5]. The availability of chitosan, its affordable cost, non-toxicity, biocompatibility, and biodegradability justify its use in sensitive applications in the biomedical and food industries. Considering food poisoning and waste, two major issues in the food industry, mainly due to microbial contamination or simply an expired shelf life of the product, using active packaging to prevent microbial contamination and the spoilage of food products and consequently extend their shelf life is of major interest for both the food industry and consumers [6,7,8]. When dissolved in weakly acidic solutions, chitosan has a high density of positive charges due to protonation of its amine functions. This unique characteristic gives rise to many interesting properties among which are a hypocholesterolemic effect, plant defense stimulation, gel formation ability, antioxidant, antiproliferative, antifungal, antibacterial, antiviral, and insecticidal activity [9]. Several studies dating from 1980 have demonstrated the antimicrobial properties of chitosan and its derivatives, with the majority focusing on chitosan solutions and films [10,11,12,13,14]. In their review article, Camacho-Martinez et al. [15] highlighted that there are very few published studies on the antimicrobial properties of chitosan nanofibers and that further investigation in this area will be of great utility for potential applications as bioactive nanomaterials. On the other hand, the main drawback of chitosan is its poor processability. According to Matet et al. [16], chitosan shows a degradation temperature lower than its melting point, which prevents the production of chitosan casted films on a large scale and their development in several applications. Furthermore, potential applications of chitosan solutions and films are limited due to poor mechanical and barrier properties.

Electrospinning of chitosan in the form of nanofibers is a promising process that has attracted much interest lately and has been the subject of recent studies [17,18,19,20,21,22,23]. The high surface area to weight ratio of the nanofiber mats, their biocompatibility, porosity, small diameter—similar to collagen fibers—and their functional properties make them particularly attractive for various applications such as tissue engineering [24], wound dressings [25], controlled drug release and gene delivery [26], water filtration [27], enzyme immobilization [28], as well as biosensors in the scope of diagnosis [24].

Three possible mechanisms of action have been proposed in the literature to explain the bactericidal activity of chitosan solutions [29,30,31,32,33]. (i) The first mechanism is related to the electrostatic attractions between the positive charges carried by chitosan chains and the negative ones present on the bacterial cell wall. Thus, low and medium molecular weight chitosan can damage the cell membrane through disruption and even perforation, causing the leakage of intracellular components and leading to bacterial lysis and consequently cell death; (ii) The second mechanism suggests that high molecular weight chitosan can form a polymer envelope which encloses the bacterial cell, thus preventing cell exchanges and the absorption of nutrients. Some authors also claimed that in the case of *E. coli*, the predominant mechanism was the first, while for *S. aureus* the second mechanism seemed more likely [34]; (iii) According to the third mechanism, the chelating effect of chitosan is involved in its antibacterial activity. Chitosan would capture trace metals and oligoelements which are essential for bacterial growth, leading to subsequent destabilization of their homeostasis. Other possible mechanisms of action have been proposed in the literature but have been considered as low probability and to be a consequence of one of the aforementioned mechanisms.

Even though the antimicrobial properties of chitosan solutions have been widely reported, the antibacterial activity of CNFs has received much less attention and has been investigated only superficially. Moreover, only a few studies have investigated the exact mechanism of action of chitosan solutions [32,35], microspheres [36], and nanocapsules [37], while CNFs’ mode of action has not been addressed yet. For example, Raafat et al. [32] have shown that lipoteichoic acid (LTA) present in Gram-positive bacteria could be involved in the first mechanism of action according to which the positive charges carried by chitosan chains can interact with the negative ones present on the bacterial membrane and cause cellular dysfunction. LTA acts therefore as a molecular link between the bacterial membrane and chitosan chains. However, LTA is a component that is present only in the cell wall of Gram-positive bacteria. Nevertheless, Gram-negative bacteria that lack it are also susceptible to the action of chitosan. Hence, the mechanism underlying chitosan’s antibacterial activity and the mode of action by which it inhibits or kills bacteria is a complex phenomenon that has not been fully explained and deserves further investigation [15].

There is considerable controversy in the literature regarding the susceptibility/resistance of Gram-positive and Gram-negative bacteria, to determine whether one or the other is more or less sensitive to the action of chitosan [29,31,34,38,39,40,41]. Hence, it has been established that this difference in strain susceptibility is likely due to structural differences in the bacterial membrane of Gram-positive and Gram-negative bacteria. However, little information is available regarding the involvement of bacterial membrane hydrophobicity/hydrophilicity, surface charge density, as well as pathogenicity in the susceptibility or resistance of both bacterial types.

This study is of great importance for the potential use of CNFs in the food packaging industry. For instance, as CNFs would be in direct contact with the packaged food, understanding their mechanism of action becomes a critical element in the fight against food spoilage and poisoning. In the present work, we examine the mechanism of action of CNFs against food spoilage *Escherichia coli* and *Listeria innocua* and pathogenic *Staphylococcus aureus* and *Salmonella* Typhimurium bacteria, under standardized conditions that mimic real food systems. We also investigate the susceptibility of Gram-positive and Gram-negative bacteria in terms of bacterial type, surface charge density, strain hydrophilicity, as well as pathogenicity. A plausible mechanism of action as well as an explanation regarding the susceptibility/resistance of bacterial strains to CNFs is proposed. To our knowledge, this study is the first that deeply investigates the mechanism of action of CNFs and their bactericidal efficiency in real conditions against meat contamination. The obtained results in terms of the antibacterial activity of CNFs are promising for their utilization as part of the active packaging materials in the scope of food protection and more specifically in meat quality preservation and shelf life extension. Another potential application is the direct use of CNFs as antimicrobial wound dressings to prevent skin infections, which has been the subject of another study [25].

## 2. Results and Discussion

In order to maximize the dose-dependent bactericidal effect of CNFs, it was necessary to use the maximum permissible content of chitosan. The 90/10 (*w/w*) chitosan/poly(ethylene oxide) CS/PEO formulation generated smooth and homogeneous nanofibers. However, the yield was not efficient because of instabilities (jet fragmentation) that took place during the electrospinning process. On the other hand, CS/PEO nanofibers with ratios less than or equal to 70/30 (*w/w*) showed a weaker antibacterial activity. Therefore, this formulation (CS/PEO 80/20) was a compromise between the 90/10 ratio that showed the highest antibacterial activity but a low yield of electrospun nanofibers, and the 70/30 ratio which exhibited a lower bactericidal effect but a higher yield. For the aforementioned reasons, the CS/PEO 80/20 formulation was selected for further characterization and analysis. 

### 2.1. Morphology of Electrospun Chitosan Nanofibers

**Figure 1:**
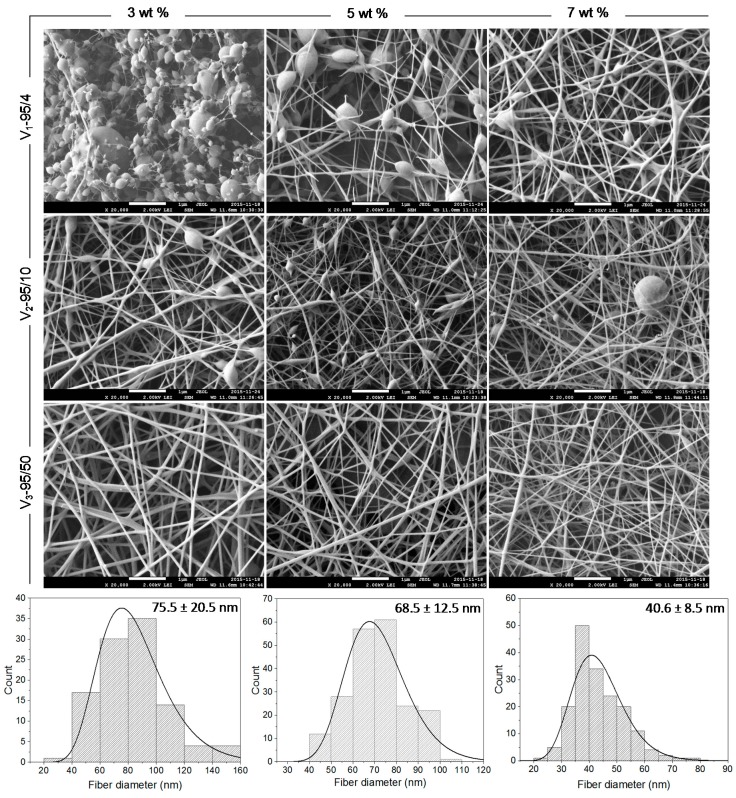
Morphology of electrosprayed and electrospun V_1_, V_2_, and V_3_ chitosan nanofibers (CNFs) and fiber diameter distribution of V_3_ CNFs. Chitosan’s concentrations: 3, 5, and 7 wt % in 50% (*v/v*) acetic acid (AcOH); Chitosan/poly(ethylene oxide) (CS/PEO) weight ratio: 80/20. All scale bars represent 1 µm.

Figure 1 presents the effect of molecular weight (*M*_W_) and concentration on the morphology of the electrospun CNFs and their related fiber diameter distributions. The results revealed that the polymer concentration is the key parameter predicting the final morphology and controlling either fiber or particle formation, regardless of the CS/PEO ratio. Our results also demonstrated that at low polymer concentrations, the molecular adhesion between chitosan chains was weak, which leads to electrospraying of the solutions and accordingly to bead formation. When the polymer concentration or *M*_W_ increased, allowing sufficient chain entanglement to form a stable filament and prevent its fragmentation, uniform and beadless nanofibers were successfully obtained (Figure 1), as also found by Pakravan et al. [17]. Indeed, the minimum concentration required for the formation of continuous and defect free nanofibers depends on a certain polymer concentration (or a multiple of it) which is known as the critical concentration of entanglement (*C_e_*) [17]. *C_e_* is significantly affected by *M*_W_ and polymer type (neutral vs. charged, i.e., flexible vs. stiff, respectively). Nevertheless, for the particular chitosan grade of 57 kDa *M*_W_ and 95% degree of deacetylation (DDA), which is close to the one used in this study (V_3_-95/50), Ardila et al. [42] reported a *C_e_* value of 2.5% (*w/v*). Moreover, McKee et al. [43] found that for neutral polymers, beaded nanofibers start to form at *C_e_*, whilst continuous and defect free nanofibers appear between 2 and 2.5 times *C_e_*. These values reach 8 to 10 times *C_e_* in the case of charged polymers such as chitosan. However, due to the difficulty of achieving such concentrations with chitosan solutions, given the high viscosity and stiffness of the system, the addition of PEO was necessary for nanofiber formation by promoting physical interactions and entanglements. Furthermore, it has been suggested that PEO can possibly interact with chitosan via hydrogen bonding [17], leading to a decrease of the electrostatic repulsions, thus decreasing the viscosity of the system while improving its flexibility and favoring fiber formation. Our results also indicated that the average fiber diameter decreased with chitosan content which was explained by an increase in electrical conductivity (data not shown). Hence, solutions with high chitosan content showed higher repulsive forces, leading to greater stretching and elongation, and consequently to nanofibers with smaller diameter and narrower fiber diameter distribution. The *M*_W_ also contributes in reaching the concentration of entanglement (*C_e_*). Indeed, for a given polymer concentration, it is known that low *M*_W_ favors bead formation. On the contrary, high *M*_W_ (longer polymer chains) enables the chain entanglement required for fiber formation. Nevertheless, a very high *M*_W_ chitosan gives rise to highly viscous and stiff systems which can be difficult or even not possible to electrospin. Overall, electrospinning is a multifactorial process and the electrospinnability of chitosan solutions is known to be severely affected by other parameters such as viscoelastic properties and surface tension of the chitosan solutions. Interestingly, the expected and final morphology of an electrohydrodynamically processed solution can be predicted and tuned by playing with the aforementioned processing and solution parameters.

### 2.2. Mechanism of Action of Chitosan Nanofibers (CNFs)—Optical Density (OD_600_)

**Figure 2:**
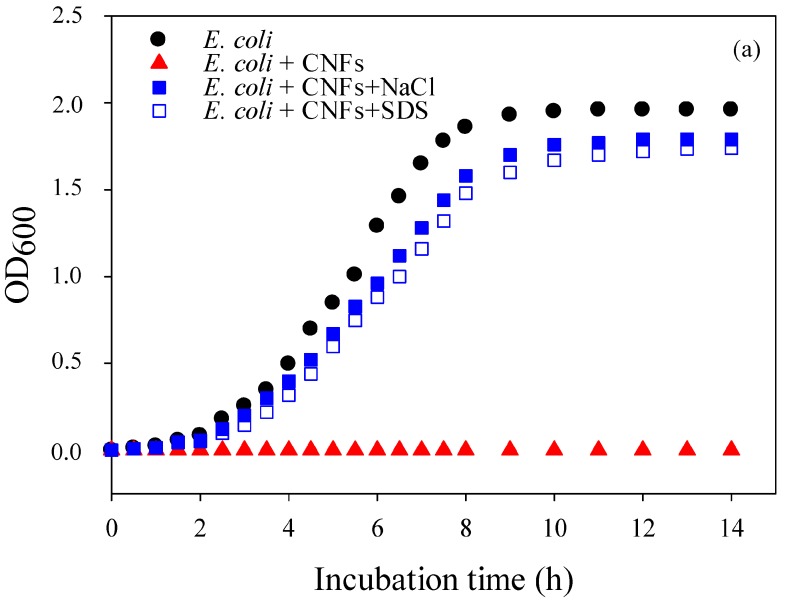

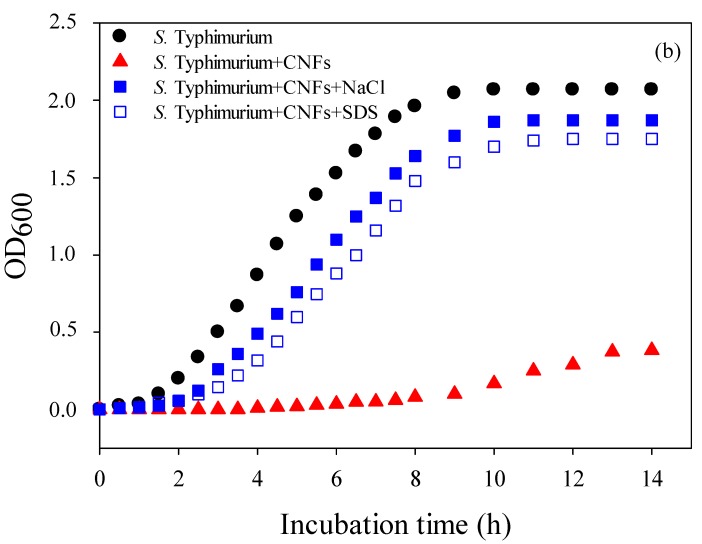
Growth curves of (**a**): *E. coli* and (**b**): *S.* Typhimurium in the absence (black circles) and in the presence (red triangles) of CNFs (2.5 cm^2^, V_3_-95/50, rich Luria-Bertani (LB) medium). Filled and empty blue squares refer to bacterial growth in contact with NaCl and sodium dodecyl-sulfate (SDS)-pretreated CNFs, respectively. The shown data are the mean values of the three replicates method.

V_3_-95/50 chitosan grade was selected for optical density (OD_600_) measurements because of its medium *M*_W_, good spinnability, and antibacterial properties, and also because this grade required the lowest concentration for fiber formation (critical entanglement concentration). Figure 2a,b, respectively, show the optical density of *E. coli* and *S.* Typhimurium cultures, in the presence and absence of CNFs. When conditions were optimal, OD_600_ resulted in a typical bacterial growth curve with the different growth phases (black curves). When the cultures were grown in the presence of CNFs, the growth of *E. coli* was completely inhibited while *S.* Typhimurium was severely altered (red curves). When the pH of the suspension was adjusted to neutrality with NaOH in order to deprotonate and inactivate chitosan, no growth recovery was observed. This suggests that the antibacterial effect was irreversible and that CNFs possess a bactericidal effect rather than bacteriostatic, as stated by other authors [32,40]. After CNFs were treated with SDS in order to screen the charges of the NH_3_^+^ groups, a visible growth with a slight decrease in OD_600_ was recorded (open blue squares), indicating that free amino groups of CNFs were responsible for the antibacterial activity. This slight decrease in optical density may be an artefact due to the lethal effect of sodium dodecyl sulfate (SDS), often used as a lysis solution at higher concentration. The decrease in OD_600_ can also be attributed to chitosan chains that can form a layer which acts as a barrier that prevents cell exchanges. However, even if proven true, it is clear that this mechanism is less intense when compared to the drastic antibacterial effect caused by the positive charges of CNFs (blue squares). When NaCl was also used to screen the positive charges on CNFs (filled blue squares), a similar effect to SDS was observed and the antibacterial activity was severely altered, allowing us to rule out the SDS lysis effect. It is important to mention that at higher salt concentrations (above 5% *w/v*) than the one used here, NaCl can also cause cell lysis of *E. coli*, as reported by Hrenovic and Ivankovic [44]. Nonetheless, the slight decrease in bacterial growth obtained with the addition of salt is probably due to the fact that some amino groups of CNFs remained protonated, which enabled a slight antibacterial activity. These results strongly indicate that the dominant mechanism of action of CNFs is attributed to their functional protonated amino groups.

### 2.3. MICs and MBCs of Chitosan in Solution State

Table 1 reports the minimum inhibitory concentrations (MICs) and minimum bactericidal concentrations (MBCs) of chitosan (CS) solutions against the tested bacteria, namely two Gram-negative and two Gram-positive model bacteria. MICs and particularly MBCs were necessary to determine the minimum concentration of chitosan that would ensure the antibacterial efficacy of the nanofibers. Our results indicate that CS significantly inhibited (MIC) or killed (MBC) the tested bacteria. However, in the case of pathogenic bacteria such as *S. aureus* and *S.* Typhimurium, the MBC that was necessary to kill 99.9% of these bacteria was 2.5 mg/mL or even higher, a concentration that coincided with the MBC of acetic acid (AcOH). Therefore, it was difficult to separate the contribution of CS from that of AcOH and determine which was responsible for the antibacterial activity. However, experiments (data not shown) conducted in water with the same CS grade revealed that the values of MBCs against *E. coli* were higher in water than in AcOH (2.5 mg/mL against 0.35 mg/mL, respectively), suggesting a synergistic effect between AcOH and chitosan.

### 2.4. Antibacterial Activity of Chitosan Nanofibers

**Figure 3:**
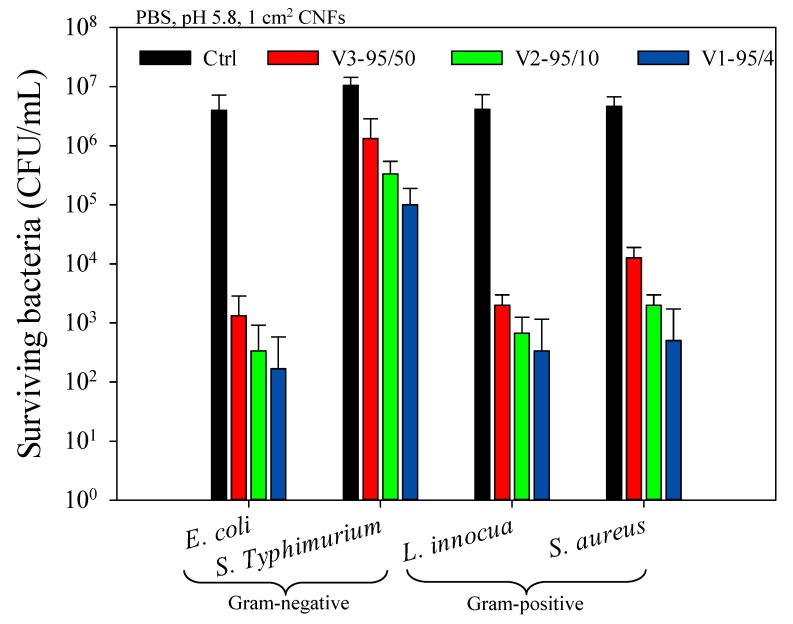
Antibacterial activity of electrospun chitosan/PEO (80/20) nanofibers with different *M*_W_ against *E. coli*, *S.* Typhimurium, *L. innocua*, and *S. aureus* after 4 h incubation in contact with CNFs.

Figure 3 shows the antibacterial activity of electrospun chitosan/PEO (80/20) nanofibers (CNFs) against *E. coli*, *S. aureus*, *L. innocua*, and *S.* Typhimurium. Overall, CNFs were very efficient in reducing and stopping bacterial growth at pH 5.8 below chitosan’s pKa. To overcome this pH dependence, quaternized chitosan could be used in order to ensure the permanent protonation of cationic sites independently from the pH of the medium [45,46]. A slightly higher effect against *E. coli* compared with *L. innocua* was observed after 4 h incubation, whilst a reduction of only 2 logs was observed for *S.* Typhimurium, which is not negligible. It is worth mentioning that, surprisingly, there was no effect of one Gram type over the other regarding susceptibility/resistance to CNFs, i.e., Gram-negative bacteria were not more or less susceptible to the action of CNFs than Gram-positive bacteria and vice-versa. More specifically, *E. coli* was significantly more susceptible compared to *S.* Typhimurium and *L. innocua* tended to be slightly more susceptible than *S. aureus* (Figure 3). The relative cell surface charge density (RCD) and hydrophilicity appear to be fundamental in understanding the difference in the sensitivities of the bacterial strains. Chung et al. [31] found that these two parameters are correlated with the inhibition efficiency of chitosan solutions (*R*^2^ = 0.942 and 0.824, respectively). Consequently, bacteria that show high RCD and hydrophilicity coefficient (hydrophilicity %) values would have a better affinity, interaction, and adsorption of chitosan chains along their cell wall, leading to a greater inhibition efficiency.

### 2.5. Kinetics of Bacterial Cell Death and Strain Susceptibility

**Figure 4:**
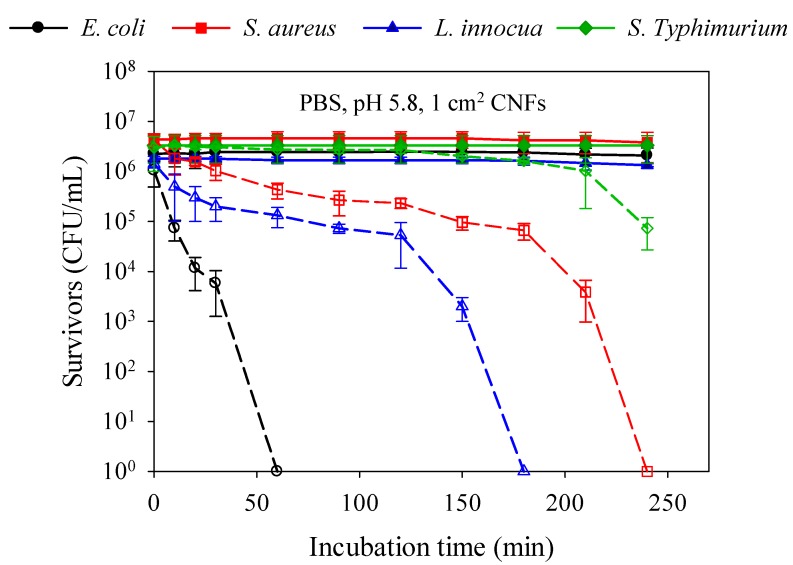
Kinetics of bacterial cell death induced by CNF (V_3_ 95/50) on Gram-negative (*E. coli* and *S.* Typhimurium) versus Gram-positive (*S. aureus* and *L. innocua*) bacteria in PBS (1×, pH 5.8) at 37 °C. Filled symbols refer to controls of bacterial suspension without treatment and empty symbols refer to the same samples after contact with CNFs.

Figure 4 presents the kinetics of bacterial cell death and the sensitivity toward CNFs (1 cm^2^ swatches) of Gram-negative (*E. coli* and *S.* Typhimurium) versus Gram-positive (*S. aureus* and *L. innocua*) bacteria at 37 °C in PBS (1×, pH 5.8). The results show that 99.9% of the Gram-negative *E. coli* were killed after 60 min of exposure, against 180 min for the Gram-positive *L. innocua*, followed by *S. aureus* (240 min), whilst a reduction of only 2 logs was observed for *S.* Typhimurium. The Gram-positive bacteria cell wall is composed of two layers: a thick peptidoglycan layer (murein) overlying the plasma membrane (the target), which consists of a single sheet lipidic bilayer. On the other hand, the cell wall of Gram-negative bacteria is composed of three layers: an outer membrane composed of a phospholipidic bilayer rich in lipopolysaccharide (LPS) and lipoproteins, a thin layer of peptidoglycan, and the inner plasma membrane. The higher hydrophilicity and negative surface charge density (SCD) of Gram-negative bacteria are thought to be mainly due to the presence of LPS [47]. Consequently, the LPS is expected to confer Gram-negative bacteria with a greater affinity to chitosan. In our study, where all the antibacterial tests were conducted in the same in vitro conditions, it was expected that Gram-negative bacteria would be more sensitive to CNFs, independently from *M*_W_, but this assumption did not apply to all Gram-negative bacteria, as observed in Figure 4. These results indicate that the antibacterial effect of CNFs is strain dependant rather than Gram dependant, and the strain sensitivity order can be listed as follows: *E. coli* > *L. innocua* > *S. aureus* > *S.* Typhimurium (Figure 4). Besides Gram type, other factors such as chitosan-bacterium interaction as well as strain pathogenicity must be taken into account.

### 2.6. Analysis of Cell Surface Hydrophobicity

**Figure 5:**
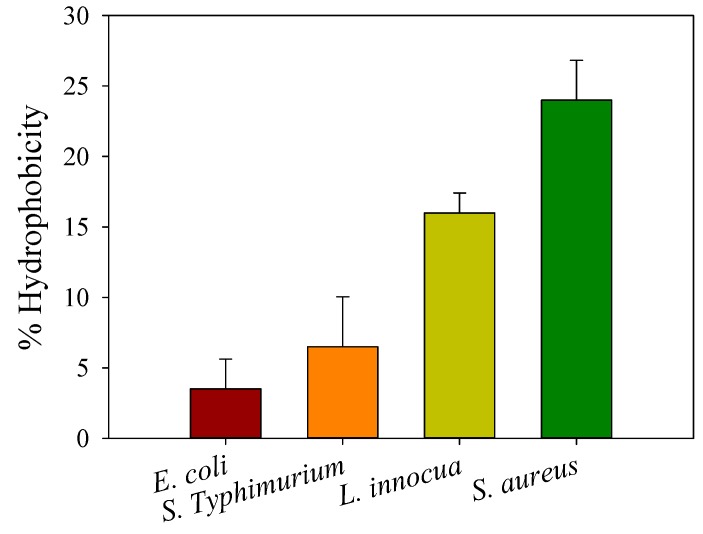
Cell surface hydrophobicity of *E. coli*, *S.* Typhimurium, *L. innocua*, and *S. aureus* bacteria, as estimated by the bacterial adhesion to a hydrocarbon (BATH) method.

Cell surface hydrophobicity and negative surface charge density appear to be fundamental in order to understand the sensitivity difference of the bacterial strains. Figure 5 shows the estimation of cell hydrophobicity measured by the bacterial adhesion to a hydrocarbon (BATH) method. The general tendency was that Gram-negative bacteria present a higher hydrophilicity (lower hydrophobicity) than Gram-positive ones. These results are in agreement with those of Chung et al. [31] who found that cell hydrophilicity and SCD are correlated with chitosan’s inhibition efficiency. The authors suggested that higher hydrophilicity and negative charge density of the cell surface of Gram-negative bacteria make them more sensitive to the action of chitosan solutions. Recently, some authors have investigated the possible involvement of the LPS in the mode of action of a synthetic aminopeptide (AMP) NK-2 against *E. coli* and *Proteus mirabilis* [48]. Since the LPS containing membrane is the first barrier of Gram-negative bacteria, the authors found that the AMP bound to and intercalated into LPS bilayers, and subsequently induced heterogeneous lesions in bacterial membranes, suggesting that the secondary targets of NK-2 are intracellular structures, such as DNA. The outer membrane of Gram-negative bacteria is mainly rich in lipopolysaccharides containing phosphate and carboxylic groups, giving the surface a high polar character, hydrophilicity, and density of negative charges in comparison with Gram-positive bacteria [47]. It is then expected that species showing high SCD and hydrophilicity values would have a better affinity, interaction, and adsorption of chitosan chains along their cell wall, leading to greater inhibition efficiency. The expected antibacterial activity should therefore be higher for all Gram-negative bacteria. However, that was not the case and strain susceptibility did not coincide with the hydrophilicity order, as shown in Figure 5. Another parameter that may be involved is the pathogenicity of the bacteria. Both *E. coli* and *S.* Typhimurium are Gram-negative but *E. coli* is innocuous while *S.* Typhimurium is pathogenic. The same observation was seen for the two Gram-positive *L. innocua* and *S. aureus* that were investigated here; the first is innocuous while the second is pathogenic. This indicates that hydrophilicity and surface charge density may explain the differences in susceptibility as the strains are innocuous. When pathogenicity is involved, bacteria show resistance toward chitosan, in the same way that some bacteria do not have the same response and show resistance to common antibiotic treatments. Currently, no other satisfactory explanation regarding the observed resistance of *S.* Typhimurium can be given. Chitosan might not be internalized in pathogenic bacteria because of recognition and/or degradation mechanisms. Indeed, further investigation of this behaviour is needed.

### 2.7. Inhibitory Activity of Chitosan Nanofibers

**Figure 6:**
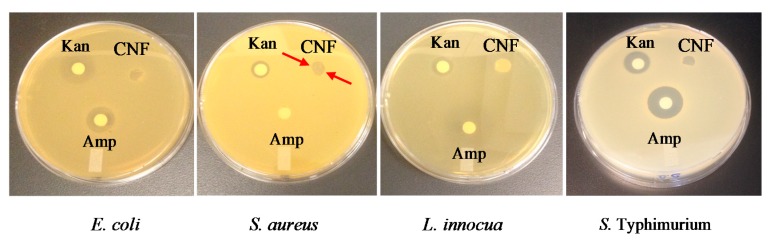
Antibiogram (Inhibition effect) of CNFs compared to kanamycin (Kan) and ampicillin (Amp) antibiotics, against *E. coli*, *S. aureus*, *L. innocua*, and *S.* Typhimurium. The arrows indicate the inhibition zone caused by chitosan.

Figure 6 shows the inhibitory activity of CNFs in comparison with two antibiotics, namely kanamycin (Kan) and ampicillin (Amp). CNFs markedly inhibited the growth of the tested bacteria as shown by the inhibition zone inside the nanofiber disks. However, no inhibition area was observed around the disks, in opposition to the two antibiotics. Chitosan did not seem able to diffuse on the agar and form that lysis area around the discs. The high *M*_W_ of chitosan in comparison with that of small molecules such as antibiotics may prevent the diffusion of its active sites through the agar. Table 2 shows that CNFs nevertheless inhibited the growth of all the tested microorganisms, namely the non-pathogenic bacteria *E. coli* and *L. innocua* and the pathogenic bacteria *S. aureus* and *S.* Typhimurium. Table 2 also indicates that the inhibitory effect increased after etching out the PEO from the mats (CNF-PEO sample), thus maximizing the chitosan-bacteria contact. It is important to note that solvent cast chitosan (CS) films (obtained by the evaporation of acetic acid) showed no inhibitory effect on bacterial growth. This is probably due to the greater surface contact area and porosity provided by the nanofibers, which suggests a better bioavailability and adsorption of chitosan functional groups to the bacterial cell membrane. In general, the inhibitory effect of CNFs was nevertheless lower than that of the antibiotics kanamycin and ampicillin. However, the inhibitory power of CNFs against the growth of *S. aureus* was higher than that of Amp, as judged by the higher inhibition zone (6 mm against 0 mm, respectively). It is therefore important to note that *S. aureus* was ampicillin-resistant but chitosan-sensitive. These results suggest that CNFs can be used as potential antibacterial coatings for medical applications, such as wound dressing, implantable medical devices, surgical suture, catheters, contact lenses, and food packaging materials, especially where bacterial development is critical to consumers’ health [25,49,50].

### 2.8. CNFs as Active Food Packaging Materials against Meat Contamination

The in situ antibacterial potential of CNFs to extend shelf life and prevent meat contamination by *E. coli* was assessed under refrigeration conditions at 4 °C (Table 3). The bacterial initial concentration (inoculum) was 2.5 × 10^3^ colony forming units per milliliter (CFU/mL). The results revealed that when contaminated meat was wrapped in a CNF plus a commercial packaging (MBP-CNFs), bacterial viability was reduced by 92%. Knowing that the initial bacterial concentration used to inoculate the meat was 2.5 × 10^3^ CFU/mL, it is evident that bacteria, fed by the nutrients present in the meat, increased in concentration. This concentration increased by one log order of magnitude and reached 2.5 × 10^4^ and 10^4^ CFU/mL in negative (MB-Ctrl^−^) and positive (MBP-Ctrl^+^) controls, respectively (Table 3). This increase in initial bacterial population was moderate in the positive control, when the samples were wrapped with the conventional packaging in comparison with the unpackaged sample (MB-Ctrl^−^). This effect was attributed to the good barrier properties provided by the commercial meat packaging which prevented the diffusion of gazes such as oxygen and water vapor, two factors that are essential to bacterial growth. Consequently, further alteration of the meat was limited and slightly slowed down. However, this type of passive packaging was unable to eliminate the bacteria initially present in the sample. PEO nanofibers (sample labelled MBP-PEONFs) were also tested and revealed to be ineffective in inhibiting the growth of *E. coli*. In contrast, CNFs, as part of the active food packaging, eradicated more than 90% of bacterial population, which enabled preservation of the microbiological quality and safety of the meat and prolonged its shelf life by 7 days at 4 °C.

## 3. Materials and Methods

### 3.1. Materials

Three water-soluble chitosan (CS) grades (Venzym™ grade) with different molecular weights and a narrow *M*_W_ distribution—obtained via enzymatic treatment of chitin—were generously donated by Ovensa Inc. (Aurora, ON, Canada). The various grades are listed in Table 4, along with the corresponding nomenclature, *M*_W_, and degree of deacetylation (DDA), provided by the supplier. Poly(ethylene oxide) (PEO) with a *M*_W_ of 600 kg/mol and glacial acetic acid (AcOH, 99.7%) were also purchased from Fisher Scientific (Ottawa, ON, Canada).

### 3.2. Methods

#### 3.2.1. Solution Preparation

Chitosan and Poly(ethylene oxide) solutions were individually prepared at concentrations of 7% and 3% (*w/v*), respectively, in 50% (*v/v*) acetic acid. Because of its good spinnability, hydrophilic character, and biocompatibility, PEO was used as a co-spinning agent to improve the spinnability of chitosan, as reported in previous studies [17,51,52]. Solutions were stirred using a magnetic stirrer for 24 h at room temperature to ensure complete dissolution of the polymer chains. To prepare CS/PEO blends, the solutions were mixed overnight at different blending ratios (50/50, 60/40, 70/30, 80/20, and 90/10).

#### 3.2.2. Electrospinning

**Figure 7:**
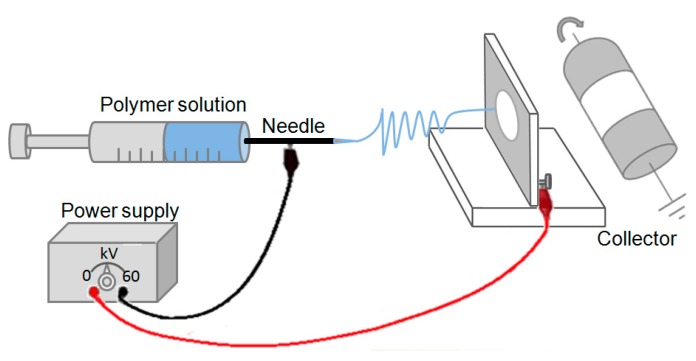
Schematic representation of the home-made electrospinning set-up.

Electrospinning was performed at room temperature according to Pakravan et al. [17] using a home-made horizontal set-up as shown in Figure 7. The solutions were poured into a 10 mL syringe connected to an 18 gauge metal needle. The syringe was placed in a programmable pump (Harvard Apparatus, PHD 2000, Saint Laurent, QC, Canada) to deliver the required CS/PEO polymer solutions. The metallic syringe was connected to a high voltage power supply (Gamma High Voltage Research, Ormond Beach, FL, USA). A metallic plate or mandrel wrapped with aluminum foil was used to collect the nanofibers in both static and rotating conditions, respectively. The electrospinning processing conditions of CS/PEO solutions are listed in Table 5.

#### 3.2.3. Scanning Electron Microscopy (SEM)

The morphology of the electrospun chitosan nanofibers was observed with a field emission scanning electron microscope (FESEM Hitachi, JEOL JSM-7600TFE field emission gamma), operated at 2 kV, as described by others [52]. For better conductivity and to reduce electron charging effects, samples were observed as collected on an aluminum foil (without any metallic coating) after 2 h of electrospinning. The spinnability and the presence of beads were also evaluated. The average fiber diameter and fiber diameter distribution were analyzed using Image-Pro Plus^®^ software. Approximately 600 nanofibers randomly chosen from three independent samples (200 nanofibers from each sample) were used for the analysis.

#### 3.2.4. Antibacterial Tests

##### Conditions

*Bacterial strains. Escherichia coli* (DH5α), *Staphylococcus aureus* (54-73), *Listeria innocua* (ISPQ3284), and *Salmonella enterica* serovar Typhimurium (SL1344), four common foodborne and skin infectious pathogenic bacteria, provided by the laboratory of microbiology, infectiology, and immunology (Université de Montréal, QC, Canada) were used as model bacteria in this study. The strains were kept at 4 °C prior to the testing and then cultured in a broth at 37 °C for 24 h. 

*Culture media.* Luria-Bertani broth (LB) and brain heart infusion (BHI) were used as growing media to start the bacterial cultures. Minimum inhibitory concentrations and minimum bactericidal concentrations of chitosan were determined against the targeted bacteria. LB agar, Muller Hinton agar, and BHI supplemented with agar (15 g/L) were used as solid media for agar plate counting.

*Inoculum.* Two colonies from the agar plate were re-suspended in 5 mL LB or BHI. The culture was then vortexed and incubated for 24 h at 37 °C under stirring in an orbital incubator shaker (New Brunswick). The final bacterial concentration was approximately 10^9^ colony forming units per millilitre (CFU/mL). To achieve 10^6^ or 10^3^ CFU/mL, bacterial cultures were diluted with a phosphate buffer saline (PBS).

##### Optical Density (OD_600_)

Optical density at 600 nm wavelength (OD_600_) using Spectrotonic 200 equipment (Thermo Fisher, Waltham, MA USA) was measured to examine the mechanism of action of chitosan nanofibers (CNFs) and their antibacterial effect on the growth of *E. coli* and *S.* Typhimurium over 24 h at 37 °C. The concept of this method is a measure of turbidity based on the Beer-Lambert law. For this purpose, sodium dodecyl sulfate (SDS 0.01 *v/v* %), an anionic surfactant, and sodium chloride (NaCl 0.5 M) were used to neutralize CNFs and screen their positive charges. All the experiments were carried out in triplicate and the results were expressed as mean values.

##### Cell Surface Hydrophobicity

The cell surface hydrophobicity of the tested bacteria was assessed by the bacterial adhesion to a hydrocarbon (BATH) method, as described by Li and McLandsborough [53]. Briefly, a 5 mL broth (LB) was inoculated with 50 µL from an overnight culture. The suspension was then incubated at 37 °C and allowed to grow up to an optical density of 0.5. Thereafter, 4 mL of this suspension was transferred into a 15 mL polypropylene tube (Falcon). A first measurement of optical density OD_600_ was then carried out and recorded as *Abs_t_*_0_. 500 µL of hexane were added to the suspension and the whole mixture was vortexed for one minute and then allowed to rest for one more minute. A second OD_600_ measurement was performed and recorded as *Abs_t_*_1_. Finally, the cell hydrophobicity was calculated according to Equation (1).

(1) $$\%\;Hydrophobicity=\frac{\;Abs_{t_{0}}-Abs_{t_{1}}}{Abs_{t_{0}}}\;\times 100$$

##### Minimum Inhibitory Concentrations and Minimum Bactericidal Concentrations of the Chitosan Solutions

The minimum chitosan concentrations necessary to inhibit bacterial growth (MIC) and to kill bacteria (MBC) were firstly determined by the colony-forming unit (CFU) method [54], using chitosan concentrations ranging from 0.005 to 5 mg/mL. Briefly described, the appropriate volume of inoculum (*E. coli*, *S.* Typhimurium, *L. innocua*, and *S. aureus*) was added to reach a bacterial concentration of 10^6^ CFU/mL. After 24 h incubation at 37 °C, a 10 µL droplet from each sample was deposited on top of the LB agar. Finally, the plates were incubated at 37 °C overnight (18 h) for further counting. The MIC and MBC of neat acetic acid solutions were also evaluated. All tests were performed in triplicate and the results expressed as mean values.

##### In Vitro Antibacterial Efficiency of CNFs

The antibacterial activities of various *M*_W_ CS/PEO nanofibers were evaluated against *E. coli*, *S.* Typhimurium, *L. innocua*, and *S. aureus* following the American Society for Testing and Materials (ASTM) standard for antimicrobial agents [54]. Bacteria were grown in LB and BHI broth for 24 h at 37 °C. After incubation, the initial concentration of the bacterial culture was brought from 10^9^ to 10^6^ CFU/mL by diluting the overnight culture with PBS (1×, pH 5.8). The nanofibers (1 cm^2^) were then placed into 5 mL of previously prepared bacterial suspension. A negative control of untreated bacteria suspended in PBS was also prepared in the same conditions. Hydrochloric acid (HCl 1 M) was used to adjust the pH of the samples. All the tubes were placed at 37 °C (optimal temperature for bacterial growth) for 4 h incubation in an orbital shaker. Agar plates were inoculated from each tube and then incubated at 37 °C overnight (18 h) for further numeration of survivors. All experiments were carried out in triplicate and the results were expressed as the mean values of three independent samples.

##### Inhibitory Activity of Chitosan Nanofibers

The inhibitory activity of electrospun CS/PEO nanofibers (CNFs) was evaluated by the inhibition zone diameter (IZD) or agar diffusion method (antibiogram) against the selected model bacteria, by using the slightly modified standard (Clinical and Laboratory Standard Institute—CLSI M02-A12) [55]. The IZDs of CNFs were also compared with those of two standard reference antibiotics, kanamycin and ampicillin (Kan and Amp, 3 µL and 5 µL, respectively). Neat chitosan nanofibers (obtained subsequently to the PEO washing of the mats), PEO nanofibers, and chitosan films (CS films, prepared by solvent evaporation of AcOH) were also analysed. One (1) mL overnight culture of the tested bacteria (10^6^ CFU/mL) was spread across the surface of a Muller Hinton agar (MHA) with pH adjusted to 5.8 with 1 M NaOH. Six (6) mm discs of Kan and Amp antibiotics and chitosan nanofibers and films were subsequently deposited on the surface of the agar plate. The plates were then incubated overnight (18 h) at 37 °C.

##### CNFs as Active Packaging Materials against Meat Contamination

Meat preservation tests were performed in order to assess the antibacterial activity of CNFs under real conditions. Briefly, 10 g of fresh meat cubes were cut under aseptic conditions. Samples were inoculated by a 30 s immersion in a bacterial suspension of *E. coli* (10^3^ CFU/mL) and were wrapped in CNF mats, immediately after drying. In order to compensate for the poor mechanical and barrier properties of CNFs, inoculated meat samples were also packaged in a conventional co-extruded multilayer food packaging (sample labelled MBP-CNFs; M for meat, B for bacteria, and P for packaging). The commercial multilayer packaging was composed of poly(ethylene terephthalate) (PET) and ethylene vinyl alcohol (EVOH), provided by ProAmpac (Terrebonne, QC, Canada). Samples were then sealed under vacuum and finally stored at 4 °C for further analysis. Negative MB–Ctrl^−^ and positive MBP–Ctrl^+^ controls of inoculated meat, wrapped with and without conventional packaging but without CNFs, were also prepared under the same conditions. The surviving bacteria were collected by grinding the meat cubes with a laboratory tissue grinder to separate bacteria from the surface of the meat tissues. After serial dilution, samples were spread on top of LB agar plates and incubated at 37 °C for 24 h for further counting of survivors. Finally, the reduction rate of the bacteria population was calculated according to the following equation [56]:

(2) $$R\;\left(\%\right)\;=\;\frac{N_{0}-\;N}{N_{0}}\text{×}100\;$$

where *N*_0_ and *N* are the numbers of colony forming units (CFU, before and after CNF treatment, respectively. The number of colony forming unit was determined as follows:

(3) $$\;\mathrm{CFU}/\mathrm{mL}=\frac{\;\mathrm{number}\;\mathrm{of}\;\mathrm{colonies}\;}{\mathrm{dilution}\;\mathrm{factor}\;\times \;\mathrm{volume}\;\left(\mathrm{mL}\right)\;}$$

## 4. Conclusions

This study is the first that investigates the mechanism of action of CNFs against Gram-negative and Gram-positive bacteria; including the strain susceptibility/resistance toward CNFs. Our in vitro results demonstrate that the predominant mechanism of action of CNFs is attributed to their functional protonated amino groups, regardless of bacterial type. Our results strongly indicate that susceptibility was not Gram-dependent, as stated in the literature, but strain-dependent. In addition, in contrast to what is stated in the literature, our findings show that chitosan’s irreversible antibacterial effect is bactericidal rather than bacteriostatic. The CNFs studied here were very efficient in reducing and stopping microorganism growth at pH 5.8 below chitosan’s pKa. To overcome this pH dependence, it is possible to restrict the use of CNFs to foods having an intrinsic weakly acidic pH such as milk, yogurts, cheeses, fish, and meat, whose pH acidifies as lactic acid is released during storage. The in situ antibacterial tests showed the potential of CNFs as bioactive nanomaterial barriers to meat contamination and showed their ability to maintain safety and extend the shelf life of fresh red meat by one week. However, another issue that may limit the use of CNFs as active food packaging is that their effectiveness is strictly conditional on contact with the packaged food, narrowing the potential applications to vacuum packaging of food products such as fresh meat, sausage, charcuteries, chicken skewers, ribs, smoked meat and salmon, fish, etc. To overcome this issue, it may be envisaged to combine the antibacterial action of CNFs with that of certain essential oils for a synergistic effect. Overall, the extension of the expiration date of unprocessed and preservative-free foods could facilitate the logistics of the whole production chain including distribution and storage, while ensuring the quality and safety of the packaged product for consumers.

## Figures and Tables

**Table 1:** Minimum inhibitory concentrations (MICs) and minimum bactericidal concentrations (MBCs) of neat AcOH and CS solutions dissolved in aqueous AcOH with concentrations ranging from 0.005 to 5 mg/mL. MICs and MBCs (mg/mL) were determined by the colony forming unit (CFU) method, after 24 h incubation at 37 °C in LB, against the four tested bacteria.

	*E. coli*		*S.* Typhimurium		*L. innocua*		*S. aureus*	
Samples	MIC *	MBC *	MIC	MBC	MIC	MBC	MIC	MBC
AcOH	0.50	2.50	2.00	>2.50	0.50	2.50	1.50	2.50
V_1_-95/4	0.05	0.15	0.15	≥2.50	0.05	0.15	0.20	0.30
V_2_-95/10	0.10	0.30	0.35	≥2.50	0.15	0.30	0.30	0.40
V_3_-95/50	0.15	0.35	0.50	≥2.50	0.25	0.40	0.40	≥2.50

* Results were expressed as mean values of three independent samples and standard deviations represented less than 7% of MIC and MBC absolute values.

**Table 2:** Inhibition zones (mm) of chitosan disks compared to kanamycin and ampicillin antibiotics against *E. coli*, *S. aureus*, *L. innocua*, and *Salmonella* Typhimurium.

Tested Discs	Inhibition Zone Diameter (mm)			
	*E. coli*	*S. aureus*	*L. innocua*	*S.* Typhimurium
CNF	6 ± 0.1	6 ± 0.1	6 ± 0.1	6 ± 0.1
CNF-PEO*	9 ± 0.3	7 ± 0.1	8 ± 0.1	7 ± 0.2
PEO NF	0 ± 0.0	0 ± 0.0	0 ± 0.0	0 ± 0.0
CS film	0 ± 0.0	0 ± 0.0	0 ± 0.0	0 ± 0.0
Kanamycin	22 ± 0.4	9 ± 0.1	10 ± 0.2	16 ± 0.2
Ampicillin	18 ± 0.3	0 ± 0.0	24 ± 0.5	19 ± 0.3

CNF-PEO*: refers to CNFs after etching out the PEO by washing with water.

**Table 3:** Antibacterial efficiency of CNFs against meat contamination by *E. coli*, after 7 day storage at 4 °C. Initial bacterial concentration was 2.5 × 10^3^ CFU/mL.

Samples	Ctrl^−^ (MB)	Ctrl^+^ (MBP)	MBP-PEONFs*	MBP-CNFs
Surviving bacteria (CFU/mL)	2.5 × 10^4^ ± 0.3	1.0 × 10^4^ ± 0.1	1.5 × 10^4^ ± 0.4	2.0 × 10^4^ ± 0.1
Reduction rate (%)	-	0.0	0.0	92.2

MBP-PEONFs*: Inoculated meat sample packed in neat PEO nanofibers (PEONFs) plus conventional packaging.

**Table 4:** Nomenclature, degree of deacetylation, and number average molecular weight (*M*_n_) of the chitosan grades used in this study.

Chitosan (Nomenclature)	DDA ^c^ (%)	*M*_n_ (kg/mol)	Company
V_1_ LMW ^a^	95	4	Ovensa
V_2_ LMW	95	10	Ovensa
V_3_ MMW ^b^	95	50	Ovensa

^a^ low molecular weight. ^b^ medium molecular weight. ^c^ degree of deacetylation.

**Table 5:** Electrospinning conditions of the CS/PEO and PEO polymer solutions.

Processing Parameters	
Flow rate (mL/h)	0.5
Voltage (kV)	25
Tip-collector distance (cm)	20
Volume (mL)	1–10
Time (h)	2–20
Temperature (°C)	RT * (21)
Relative humidity (%)	7–40

* Room temperature.
